# Ronapreve (REGN-CoV; casirivimab and imdevimab) reduces the viral burden and alters the pulmonary response to the SARS-CoV-2 Delta variant (B.1.617.2) in K18-hACE2 mice using an experimental design reflective of a treatment use case

**DOI:** 10.1128/spectrum.03916-23

**Published:** 2024-07-16

**Authors:** Lee Tatham, Anja Kipar, Jo Sharp, Edyta Kijak, Joanne Herriott, Megan Neary, Helen Box, Eduardo Gallardo Toledo, Anthony Valentijn, Helen Cox, Henry Pertinez, Paul Curley, Usman Arshad, Rajith Kumar Reddy Rajoli, Steve Rannard, James P. Stewart, Andrew Owen

**Affiliations:** 1 Department of Pharmacology and Therapeutics, Institute of Systems, Molecular and Integrative Biology, University of Liverpool, Liverpool, United Kingdom; 2 Centre of Excellence in Long-acting Therapeutics (CELT), University of Liverpool, Liverpool, United Kingdom; 3 Laboratory for Animal Model Pathology, Institute of Veterinary Pathology, Vetsuisse Faculty, University of Zurich, Zurich, Switzerland; 4 Department of Infection Biology & Microbiomes, Institute of Infection, Veterinary and Ecological Sciences, University of Liverpool, Liverpool, United Kingdom; 5 Department of Chemistry, University of Liverpool, Liverpool, United Kingdom; Karolinska Institutet, Stockholm, Sweden

**Keywords:** SARS-CoV-2, mAb, preclinical PK/PD

## Abstract

With some exceptions, global policymakers have recommended against the use of existing monoclonal antibodies in COVID-19 due to loss of neutralization of newer variants. The purpose of this study was to investigate the impact of Ronapreve on compartmental viral replication using paradigms for susceptible and insusceptible variants. Virological efficacy and impact on pathogenicity was assessed in K18-hACE2 mice inoculated with either the Delta or BA.1 Omicron variants. Ronapreve reduced sub-genomic viral RNA levels in lung and nasal turbinate, 4 and 6 days post-infection, for the Delta variant but not the Omicron variant. It also blocked brain infection, which is seen with high frequency in K18-hACE2 mice after Delta variant infection. At day 6, the inflammatory response to lung infection with the Delta variant was altered to a multifocal granulomatous inflammation in which the virus appeared to be confined. The current study provides evidence of an altered tissue response to SARS-CoV-2 after treatment with a monoclonal antibody combination that retains neutralization activity. These data demonstrate that experimental designs that reflect treatment use cases are achievable in animal models for monoclonal antibodies. Extreme caution should be taken when interpreting prophylactic experimental designs that may not be representative of treatment.

**IMPORTANCE:**

Following the emergence of the SARS-CoV-2 Omicron variant, the WHO recommended against the use of Ronapreve in its COVID-19 treatment guidelines due to a lack of efficacy based on current pharmacokinetic-pharmacodynamic understanding. However, the continued use of Ronapreve, specifically in vulnerable patients, was advocated by some based on *in vitro* neutralization data. Here, the virological efficacy of Ronapreve was demonstrated in both the lung and brain compartments using Delta as a paradigm for a susceptible variant. Conversely, a lack of virological efficacy was demonstrated for the Omicron variant. Comparable concentrations of both monoclonal antibodies were observed in the plasma of Delta- and Omicron-infected mice. This study made use of a reliable murine model for SARS-CoV-2 infection, an experimental design reflective of treatment, and demonstrated the utility of this approach when assessing the effectiveness of monoclonal antibodies.

## INTRODUCTION

Since the emergence of severe acute respiratory syndrome coronavirus-2 (SARS-CoV-2) in late 2019, a concerted global effort resulted in a toolbox of putative interventions that were brought through development at unprecedented speed. The rapid development and implementation of vaccination programs have had a transformational impact on control of the pandemic in some countries, but ongoing efforts for vaccine equity continue to be critical (1). In addition, first-generation antiviral drugs have emerged from repurposed small molecules from other antiviral development programs, such as drugs originally developed for Ebola, influenza, or prior coronaviruses. More potent antivirals continue to emerge, but considerable research is still required to optimize the deployment of existing agents (including evaluation of regimens composed of drug combinations) (2, 3).

Neutralizing monoclonal antibodies targeting the spike protein on the surface of SARS-CoV-2 were also brought forward with commendable speed, but the urgency of the pandemic necessitated that key knowledge was not collected during the accelerated development process. Ronapreve (REGN-COV2) is composed of two such monoclonal antibodies (casirivimab and imdevimab) and demonstrated clinical efficacy against pre-Omicron SARS-CoV-2 variants in post-exposure prophylaxis (4), early treatment (5), and in the treatment of seronegative patients with severe coronavirus disease 2019 (COVID-19) (6). With the successive emergence of Omicron sub-lineages, all approved monoclonal antibodies have lost varying degrees of neutralization capability such that continued efficacy in all use cases is no longer plausible based upon the current understanding of the pharmacokinetic-pharmacodynamic relationship (7–9). As a result, no monoclonal antibodies are currently recommended by the NIH or WHO (10–12). However, the continued use of Ronapreve in vulnerable patients is advocated by some based on *in vitro* neutralization data for Omicron lineages (13). Each antibody in Ronapreve exhibits molar potency against previous SARS-CoV-2 variants, which are orders of magnitude higher than current repurposed small molecule drugs such as molnupiravir and nirmatrelvir (10, 14), but they were given in combination to reduce the risk of emergence of resistance as has been widely documented for monoclonal antibodies used against susceptible variants as monotherapy (15–21).

Several variants of concern (VOC) have emerged over the past 2 years to which at least one of the antibodies in Ronapreve has retained *in vitro* activity albeit at a lower potency than against the ancestral virus (22). Moreover, the efficacy of Ronapreve against the Delta variant was demonstrated across various clinical trials (23–26). Studies in K18-hACE2 transgenic mice clearly demonstrated the virological efficacy of Ronapreve against previous variants (not including Delta, which was not studied) (27). Several studies have also investigated the activity of casirivimab and imdevimab (alone or in combination) against pseudovirus engineered to express the BA.1 Omicron spike protein or authentic virus (28–30). All studies have demonstrated compromised activity of the Ronapreve combination in these assays. However, other studies reported residual activity of the individual antibodies when studied in isolation, albeit with substantially lower activity (31). Unlike other monoclonal antibodies, extremely high doses of casirivimab and imdevimab (up to 8,000 mg intravenously) have been studied safely, and pharmacokinetics at these doses far exceed stringent target concentrations developed by the manufacturers for ancestral SARS-CoV-2 (5).

Recent studies provided evidence that intraperitoneal administration of neutralizing human antibodies protect K18-hACE2 mice from lung infection and clinical disease in both prophylactic (hours to 3 days prior to intranasal infection, using an ancestral SARS-CoV-2 and the Delta variant) and therapeutic settings (up to 4 days post-intranasal infection) (32, 33). A study using neutralizing murine monoclonal antibodies demonstrated a significant reduction of viral titers in the lungs at 2 days post-infection (dpi), i.e., the peak of lung infection in untreated mice, when mice were treated with the antibody at 6 hours post-intranasal infection. Similarly, prophylactic treatment (day −1) prior to infection with an original virus isolate significantly reduced weight loss and viral titers in nasal turbinate, lungs, and brain at 5 dpi; interestingly, treatment at 5.5 hours post-infection had the same effect on body weight and viral loads in all tested organs except the lungs (34).

The purpose of this study was to investigate the ability of monoclonal antibody combinations to mitigate pulmonary and neurological manifestations of SARS-CoV-2 infection using Ronapreve and the Delta variant as a paradigm for activity against a susceptible variant. *In vivo* validation of prior *in vitro* assay readouts for neutralization of BA.1 Omicron by Ronapreve is also presented.

## RESULTS

### Body weight

**Fig 1:**
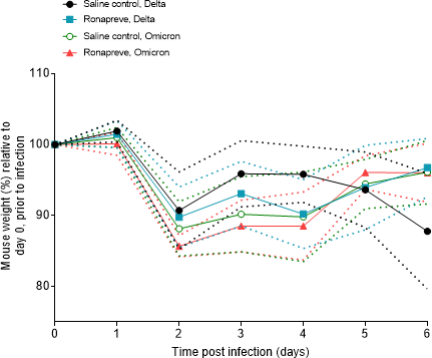
Mouse weights separated by treatment group and infection status. Weights are the percentage of the initial weight recorded at day 0 prior to infection. Standard deviations are indicated by the dashed plots.

Weight was monitored throughout the study as a marker for health. Figure 1 shows mouse weights relative to baseline (day 0; prior to SARS-CoV-2 inoculation). All animals displayed weight loss at day 2 post-infection (9.3%–14.3% of body weight); this was less rapid in the Delta-variant-infected animals, compared to the Omicron infected, albeit without statistical significance. Most animals regained some weight (2.1%–5.2%) by day 3, and most reached pre-infection levels (around 95%) by day 6 with the exception of the control Delta-variant-infected animals, which showed progressive weight loss after day 4, partly reaching the clinical endpoint (up to 20% weight loss) by day 6.

### Effect of Ronapreve on viral replication

**Fig 2:**
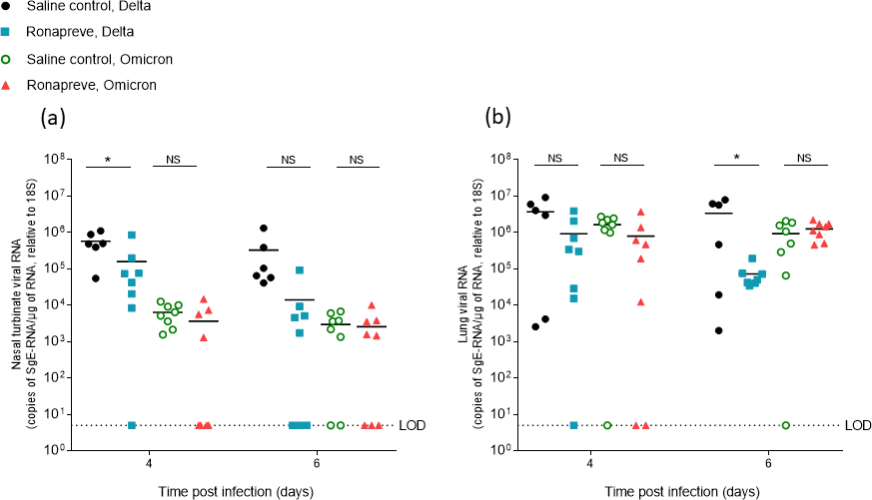
Viral quantification of SARS-CoV-2 sub-genomic RNA (sgE), relative to 18S, using qRT-PCR from nasal turbinate (**a**) and lung (**b**) samples harvested from each group on days 4 and 6 post-infection. Mice infected with the Delta variant were administered with a single IP dose of either saline (*n* = 12) or Ronapreve, 400 µg/mouse, in saline (*n* = 16). Equally, mice infected with the Omicron variant were administered with a single IP dose of either saline (*n* = 16) or Ronapreve, 400 µg/mouse, in saline (*n* = 16). Data for individual animals are shown with the mean value represented by a black line. NS, not significant; *, *P* ≤ 0.05 (unpaired, two-tailed *t*-test).

To determine the viral load in animals infected with each variant and subsequently dosed with either saline (controls) or Ronapreve, total RNA was extracted from the lung and nasal turbinate samples of animals culled on days 4 and 6 post-infection. Viral replication was quantified using qRT-PCR to measure sub-genomic viral RNA to the E gene (sgE) as a proxy. The results are illustrated in Fig. 2. In SARS-CoV-2 Delta-variant-infected animals, the amount of sgE RNA was generally reduced after Ronapreve treatment compared to the saline-treated mice. At 4 dpi, the difference was significant in the nasal turbinates (log_10_ fold decrease: −0.556, *P* = 0.037) but not in the lung (log_10_ fold decrease: −0.602, *P* = 0.065), whereas at 6 dpi, the difference was not significant in the nasal turbinates (log_10_ fold decrease: −1.369, *P* = 0.111) but significant in the lung (log_10_ fold decrease: −1.667, *P* = 0.033).

In contrast, in the Omicron-infected mice, the amount of sgE RNA detected in the nasal turbinates was only marginally reduced at both 4 dpi (log_10_ fold decrease: −0.243, *P* = 0.267) and 6 dpi (log_10_ fold decrease: −0.065, *P* = 0.973) in the Ronapreve-treated mice compared to the saline controls. The same effect was observed in the lung at 4 dpi (log_10_ fold decrease: −0.312, *P* = 0.149), whereas an increase was observed at 6 dpi (log_10_ fold increase: 0.130, *P* = 0.390). The results highlight the diminished *in vivo* antiviral potency of Ronapreve against the Omicron variant.

**Fig 3:**
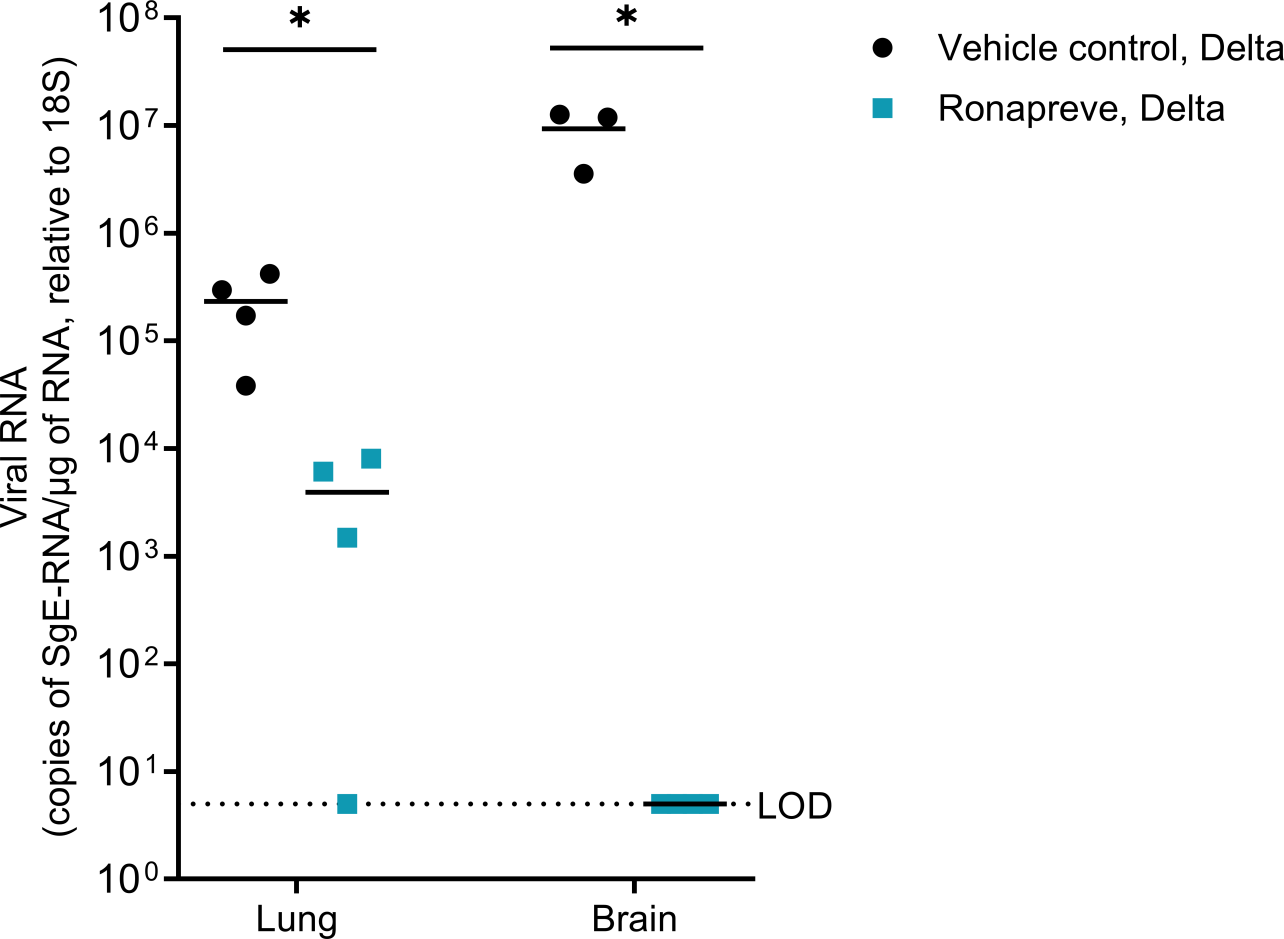
Virological efficacy of Ronapreve in K18-hACE2 mice infected with SARS-CoV-2 Delta. Viral quantification of SARS-CoV-2 sub-genomic RNA (sgE), relative to 18S, using qRT-PCR in lung and brain samples harvested from each group 9 days post-infection. Infected mice were administered with a single IP dose of either saline (*n* = 4) or Ronapreve, 400 µg/mouse, in saline (*n* = 4). Data for individual animals are shown with the mean value represented by a black line. *, *P* ≤ 0.05 (unpaired, two-tailed *t*-test). One complete brain from the saline-administered mice was used for another *in situ* study. The remaining brain samples (*n* = 3) were available for qPCR analysis.

A separate study, utilizing a comparable experimental design and the SARS-CoV-2 Delta variant, was performed to determine whether viral replication in the brain was indeed blocked by Ronapreve treatment. Sub-genomic E gene RNA (sgE) levels were quantified in both lung and brain at 9 dpi using qRT-PCR. These data confirmed viral replication in the lungs of all animals and in the brain of vehicle control treated mice, whereas sgE levels were below the level of detection in the brains of the Ronapreve-treated mice. The results are shown in Figure 3. Overall, the amount of sgE RNA was significantly reduced in both the lung and brain after Ronapreve treatment compared to the vehicle control treatment. Specifically, for lung, a log_10_ fold decrease of −1.772, *P* = 0.032, and for brain, a log_10_ fold decrease of −6.272, *P* = 0.012 was observed.

### Differences in viral replication between Delta and Omicron variants

Mice were challenged with a comparable amount of virus (10^3^ PFU) of both SARS-CoV-2 variants. However, comparison of the sgE RNA levels in the tissues of the saline-treated animals at both time points showed that infection with the Omicron variant generally yielded lower viral loads (Fig. 2). In the nasal turbinate samples, a log_10_ fold lower viral RNA level of −0.243, *P* = 0.267 (4 dpi) and −2.043, *P* = 0.099 (6 dpi) was observed in the Omicron group (Fig. 2a). In the lung, a log_10_ fold lower viral RNA level of −0.353, *P* = 0.137 (4 dpi) and −0.561, *P* = 0.085 (6 dpi) was observed (Fig. 2b). Detailed information on viral loads in individual animals is provided in Table S1. Similar trends have been reported in Omicron-infected mice displaying a lower viral load in both upper and lower respiratory tracts (35).

### The effect of Ronapreve on pulmonary changes and viral spread to the brain after infection with the Delta and Omicron variants

**Fig 4:**
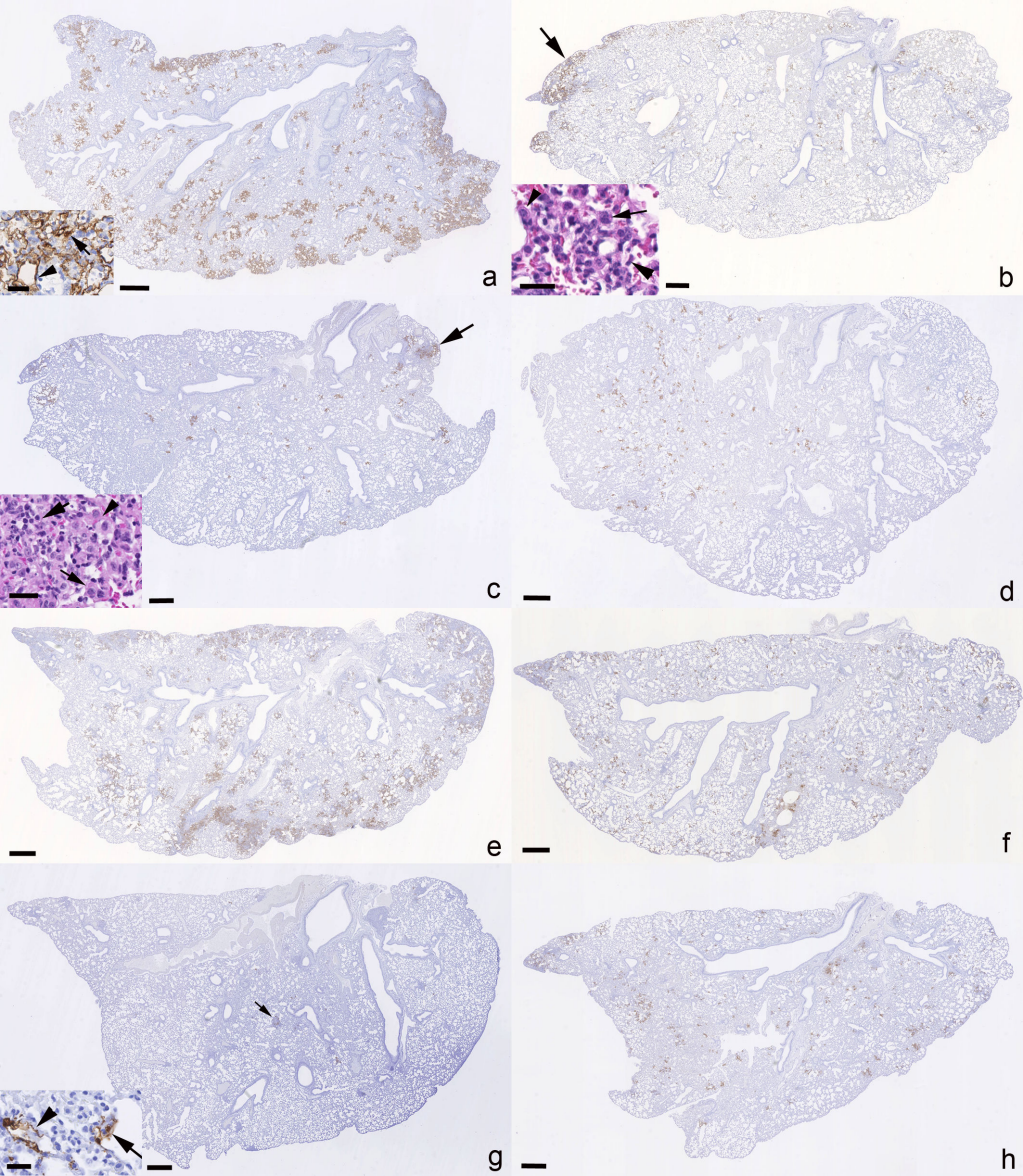
Left lung, longitudinal sections, K18-hACE2 mice. SARS-CoV-2 N expression at day 4 (**a-d**) and day 6 (**e-h**) post-infection with 10^3^ PFU of SARS-CoV-2 Delta variant (B.1.617.2; **a, c, e, g**) or Omicron variant (**b, d, f, h**), followed after 24 hours by an intraperitoneal injection of 100 µL saline control (**a, b, e, f**) or 400 µg Ronapreve (**c, d, g, h**), diluted in saline. (**a**) Delta-variant-infected mouse (C1.2) treated with saline control, 4 dpi. Abundant large, partly coalescing patches of alveoli with SARS-CoV-2 N positive epithelial cells are found disseminated throughout the parenchyma. The inset confirms infection of both type I (arrowhead) and type II (arrow) pneumocytes. (**b**) Omicron-variant-infected mouse (C2.1) treated with saline control, 4 dpi. There are multiple disseminated small patches of alveoli with positive epithelial cells. A large patch (arrow) of positive alveoli is seen in association with focal desquamation of alveolar epithelial cells (inset: large arrowhead) and the presence of activated (inset: small arrowhead) and syncytial (inset: arrow) type II pneumocytes. (**c**) Delta-variant-infected mouse (R1.5) treated with Ronapreve, 4 dpi. There are numerous small disseminated patches of alveoli with positive epithelial cells, and larger patches (arrow) in association with focal activation (inset: arrowhead) and syncytia formation (inset: small arrow) in type II pneumocytes, desquamation of alveolar epithelial cells, occasional degenerate cells, and a few infiltrating lymphocytes and neutrophils (inset: large arrow). (**d**) Omicron-variant-infected mouse (R2.6) treated with Ronapreve, 4 dpi. Viral antigen expression is seen in epithelial cells of random small patches of alveoli. (**e**) Delta-variant-infected mouse (C3.5) treated with saline control, 6 dpi. Multifocal extensive, partly coalescing large patches of alveoli with positive epithelial cells are found disseminated throughout the parenchyma. (**f**) Omicron-variant-infected mouse (C4.8) treated with saline control, 6 dpi. There are multiple disseminated, mainly small patches of alveoli with positive epithelial cells. (**g**) Delta-variant-infected mouse (R3.4) treated with Ronapreve, 6 dpi. There are disseminated very small patches of alveoli with positive epithelial cells (inset arrowhead: type I pneumocyte, arrow: type II pneumocyte). Positive cells are also observed in focal infiltrates (arrow; see Fig. 4). (**h**) Omicron-variant-infected mouse (C4.8) treated with Ronapreve, 6 dpi. There are numerous disseminated, mainly small patches of alveoli with pos epithelial cells. Immunohistochemistry for SARS-CoV-2 N, hematoxylin counterstain, and HE stain (insets in b and c). Bars = 500 µm (overviews) and 50 µm (insets).

Productive viral infection and any associated pathological changes were determined in nose and lungs of all mice at both 4 and 6 dpi and confirmed an identical spectrum of viral target cells in nasal mucosa and lung for both virus isolates, comprising respiratory and olfactory epithelial cells in the former, and both alveolar type I and II pneumocytes in lungs (Fig. 4) as previously described (36–38).

At 4 dpi, virus antigen expression was detected in nasal respiratory epithelial cells in all Delta infected but none of the Omicron-infected, saline-treated mice, which aligned with the PCR data from the nasal turbinates, showing lower sgE RNA levels in Omicron-infected mice compared to Delta-infected mice. In the lung, viral antigen was detected in five of the six Delta-infected mice. Infection was generally widespread and seen in numerous, large, partly coalescing patches of alveoli (Fig. 4a). Consistent with previous reports (38, 39), this was accompanied by the presence of activated type II pneumocytes, occasional syncytial cells and degenerate occasionally desquamed alveolar epithelial cells, increased interstitial cellularity, and mild vasculitis. In the Omicron-infected mice, viral antigen was detected in the lungs of seven of eight animals but was overall less abundant than in the Delta-infected mice. It was seen in disseminated small patches of alveoli (Fig. 4b) where it was accompanied by focal histological changes of the same nature as those seen with Delta infection (Fig. 4b inset). The lung PCR data revealed no significant difference between the saline-treated Omicron-infected mice and the Delta-infected mice (*P* = 0.137). Interestingly, animal C2.5 (Table S1) was negative for both viral antigen and sgE RNA, demonstrating consistency between the immunohistochemistry and PCR data.

After Ronapreve treatment, at 4 dpi, the nasal mucosa harbored SARS-CoV-2 N positive respiratory epithelial cells in four of the eight Delta-infected mice and in one of the eight Omicron-infected mice. The lungs of seven of the eight Delta-infected mice were found positive for viral antigen but the expression was generally less extensive than in the saline-treated group and restricted to small disseminated patches of alveoli (Fig. 4c). Infection was accompanied by similar histological changes as in the untreated mice, but these were less extensive and focal (Fig. 4c inset). In Omicron-infected mice, viral antigen was detected in five of the eight lungs, with a similar extent and distribution as in the Delta-infected mice and the untreated Omicron-infected group (Fig. 4d), and with histological changes similar to those seen in the untreated Omicron-infected mice in nature and extent.

**Fig 5:**
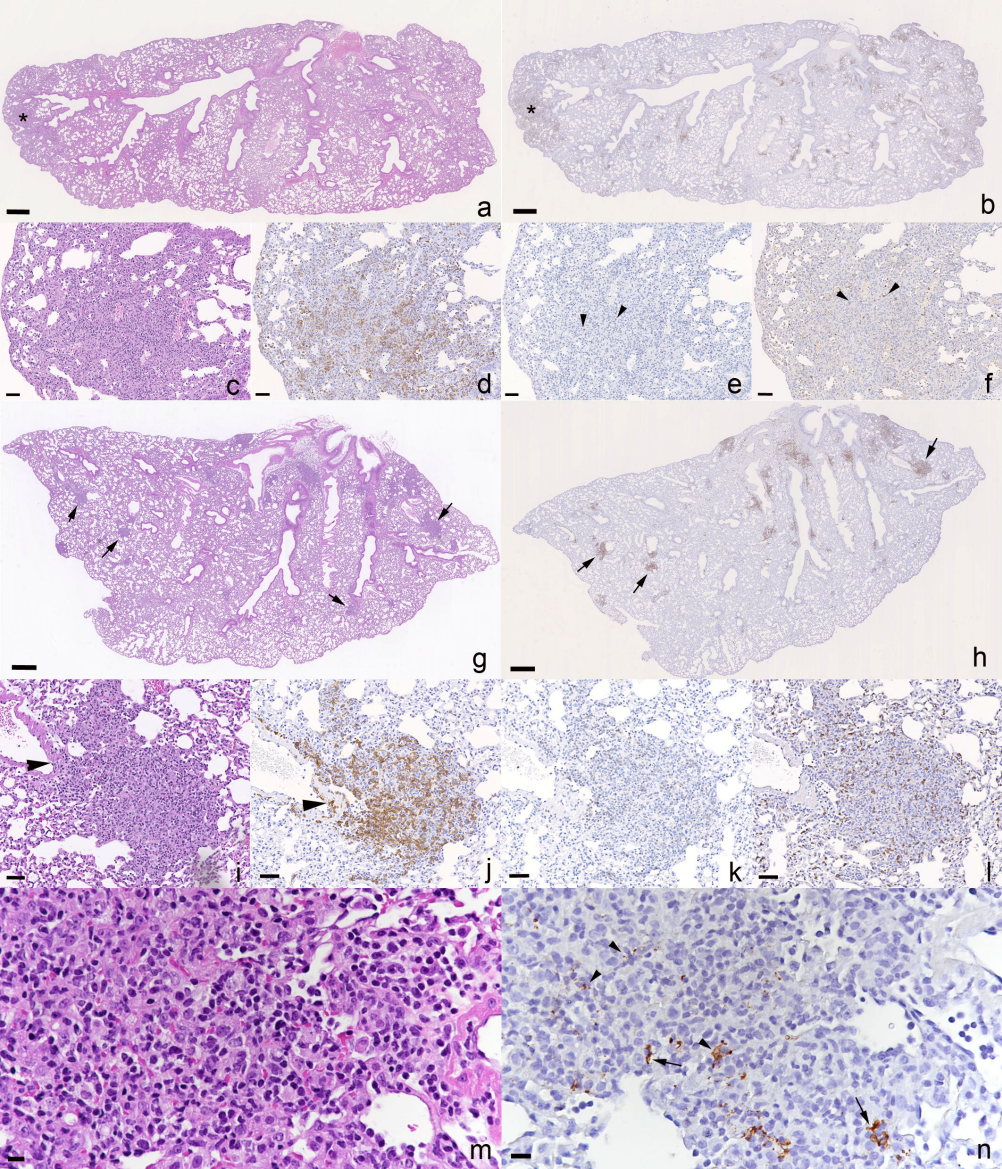
Lungs, K18-hACE2 mice at day 6 post-infection with 10^3^ PFU of SARS-CoV-2 Delta variant (B.1.617.2), followed after 24 hours by an intraperitoneal injection of 100 µL saline control or 400 µg Ronapreve, diluted in saline. (**a-f**) Saline-treated animal (C3.1). (**a**) The parenchyma shows a focal consolidated area (asterisk) and several areas with increased cellularity (arrow) in the parenchyma. (**b**) Macrophages (Iba1+) are abundant in the consolidated area (asterisk). (**c-f**) Closer view of the consolidated area confirming that macrophages (d; Iba1+) are the dominant inflammatory cells, with a few intermingled individual T cells (e; CD3+, arrowheads) and B cells (f; CD45R/B220, arrowheads). (**g-n**) Ronapreve-treated animal (R3.2). (**g**) The parenchyma exhibits several well-delineated, dense inflammatory infiltrates (arrows). (**h**) The infiltrates (arrows) appear to comprise macrophages (Iba1+). (**i-l**) Focal inflammatory infiltrate. (**j**) Macrophages (Iba1+) are the dominant inflammatory cells and are also seen to emigrate from a vessel (arrowhead). T cells (**k**) and B cells (**l**) are seen intermingled in small numbers. (**m, n**) Closer view of a focal inflammatory infiltrate. The mononuclear infiltrate (**m**) contains viral antigen (**n**) within a few pneumocytes (arrows) and cell free or phagocytosed within macrophages (arrowheads). (a, c, g, I, m) HE stain; (b, d-g, h, j-l, n) immunohistochemistry. Bars = 500 µm (**a, b, g, h**) and 25 µm (all others).

At 6 dpi, in saline-treated animals, virus antigen was still detected in respiratory epithelial cells in the nasal mucosa in all Delta-infected mice but in none of the Omicron-infected mice, which aligned with the lower sgE RNA levels in this group compared to the Delta-infected mice (Fig. 2), although the difference was not statistically significant (*P* = 0.099). In the lungs, SARS-CoV-2 antigen was detected in four of the six Delta-infected animals, mainly in numerous, often large disseminated patches of alveoli (Fig. 4e), and most intense in association with large consolidated areas of increased interstitial cellularity that contained activated type II pneumocytes, occasional syncytial cells and degenerate and/or desquamed alveolar epithelial cells, and a macrophage-dominated inflammatory infiltrate (Fig. 5a through f). In the Omicron-infected mice, viral antigen expression was detected in all eight animals, generally in numerous disseminated small patches of alveoli (Fig. 4f). It was overall less extensive than in the Delta-infected animals at this time point, supporting the virology results, with lower sgE RNA levels in the saline-treated Omicron-infected mice compared to the Delta-infected mice (Fig. 2), although the difference was not statistically significant (*P* = 0.085). Infection was accompanied by mild histological changes, represented by small focal areas with desquamed alveolar epithelial cells and mild mononuclear infiltration.

After Ronapreve treatment, at 6 dpi, there was still limited evidence of viral antigen expression in the nasal respiratory epithelium, in 6/8 Delta infected. It was also detected in the lungs but restricted to a few small patches of alveoli in Delta-infected animals (Fig. 4g), whereas it was similar in its extent to day 4 and seen as disseminated small patches of positive alveoli in all Omicron-infected mice (Fig. 4h). In the Delta-infected mice, however, multifocal small, delineated, dense parenchymal mononuclear infiltrates were consistently seen (Fig. 5g through n). These comprised macrophages (Iba1+), with lesser T cells (CD3+) and B cells (CD45R+) and also involved vessels, where a patchy vasculitis, with focal infiltration of the vascular wall, stretching into a focal perivascular infiltrate, was observed (Fig. 5g, i, and j). The lesions (Fig. 5m) often contained a few infected alveolar epithelial cells and some free viral antigen, consistent with debris of infected cells (Fig. 5n). In the Omicron-infected animals, the histological changes were generally mild and as described for the control mice, although focal infiltrates similar to those seen in the Delta-infected treated mice were also seen, albeit overall less pronounced and less delineated. Detailed information on histological findings, viral antigen expression, and viral loads in individual animals is provided in Table S1.

**Fig 6:**
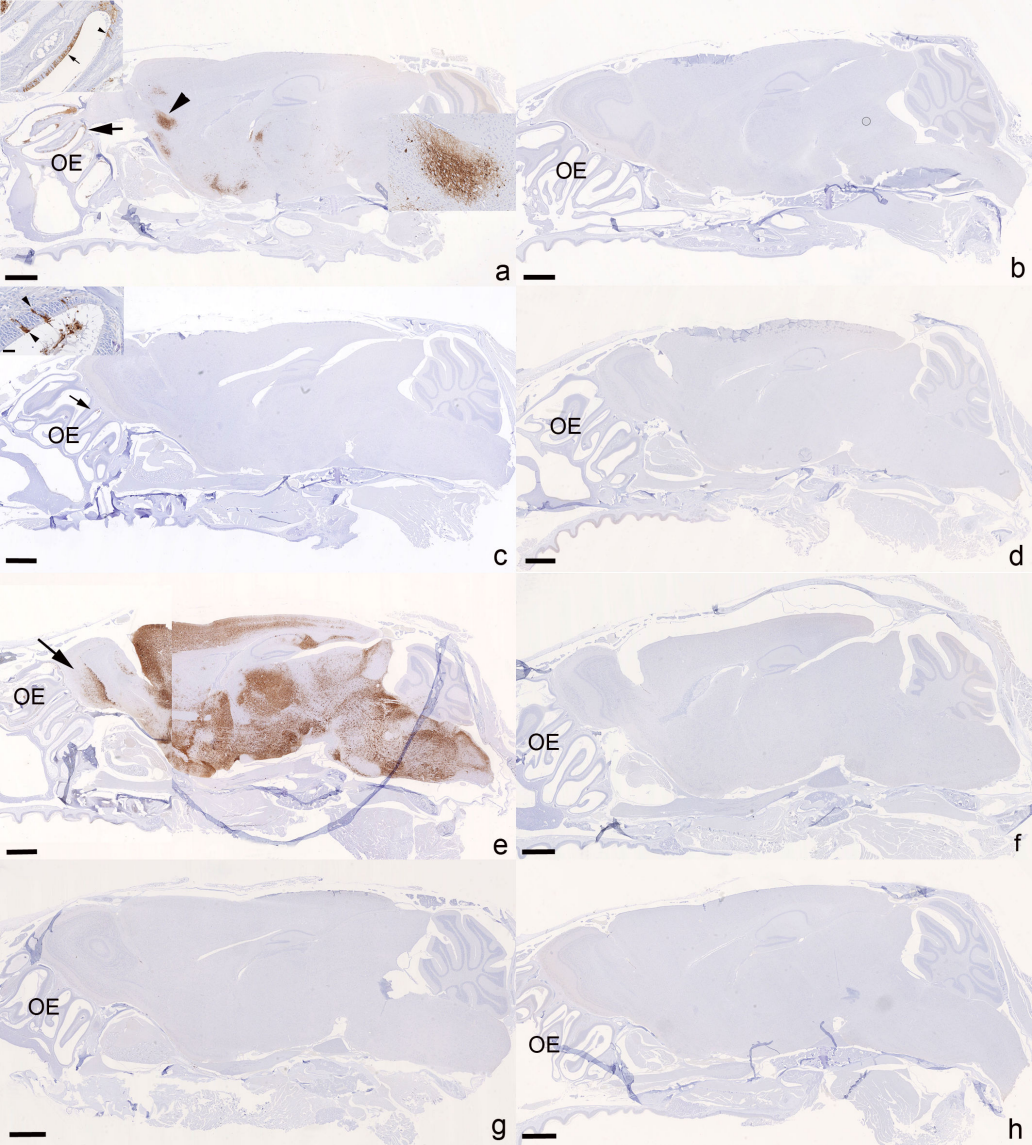
Heads with olfactory epithelium (OE) and brain, K18-hACE2 mice. SARS-CoV-2 N expression at day 4 (**a-d**) and day 6 (**e-h**) post-infection with 10^3^ PFU of SARS-CoV-2 Delta variant (B.1.617.2; **a, c, e, g**) or Omicron variant (**b, d, f, h**), followed after 24 hours by an intraperitoneal injection of 100 µL saline control (**a, b, e, f**) or 400 µg Ronapreve (**c, d, g, h**), diluted in saline. (**a**) Delta-variant-infected mouse (C1.1) treated with saline control, 4 dpi. The virus is widespread in the OE (arrow and inset showing a large patch of positive epithelial cells [arrow] and a few individual positive epithelial cells [arrowhead]) and has spread to the brain; there are patches of neurons positive for viral antigen in frontal cortex, cerebral nuclei (caudoputamen), hypothalamus/thalamus, midbrain, and pons. The arrowhead depicts a large patch of positive neurons in the frontal cortex of which a closer view is provided in the inset. (**b**) Omicron-variant-infected mouse (C2.3) treated with saline control, 4 dpi. There is no evidence of viral antigen expression in the OE and brain. (**c**) Delta-variant-infected mouse (R1.1) treated with Ronapreve, 4 dpi. There is no evidence of viral antigen expression in the brain. The OE exhibits a small patch with positive epithelial cells. Inset: OE with viral antigen expression in intact individual olfactory epithelial cells (arrowheads) and in degenerate cells in the lumen of the nasal cavity. (**d**) Omicron-variant-infected mouse (R2.5) treated with Ronapreve, 4 dpi. There is no evidence of viral antigen expression in the OE and brain. (**e**) Delta-variant-infected mouse (C3.3) treated with saline control, 6 dpi. There is widespread viral antigen expression in abundant neurons throughout the brain including the olfactory bulb (left, arrow), with the exception of the cerebellum. (**f**) Omicron-variant-infected mouse (C4.1) treated with saline control, 6 dpi. There is no evidence of viral antigen expression in the OE and brain. (**g**) Delta-variant-infected mouse (R3.3) treated with Ronapreve, 6 dpi. There is no evidence of viral antigen expression in the OE and brain. (**h**) Omicron-variant-infected mouse (C4.7) treated with Ronapreve, 6 dpi. There is no evidence of viral antigen expression in the OE and brain. Immunohistochemistry, hematoxylin counterstain. Bars = 1 mm.

We and others have previously shown that wild type and VOC SARS-CoV-2s readily spread to the brain in K18-hACEs mice; Omicron variants appear not to have the same effect, as there is no evidence of viral antigen expression in the brains (39). The current study confirmed these findings. Brain infection was a rather consistent finding in the untreated Delta-infected animals. At 4 dpi, viral antigen was detected multifocally in neurons in the brain in four of the six mice (Fig. 6a), which all exhibited infection of the olfactory epithelium (Fig. 6a inset). SARS-CoV-2 N expression was also detected in nerve fibers or a variable amount of neurons in the olfactory bulb, consistent with viral spread from the nasal mucosa, via the olfactory plate (40). There was no evidence of an inflammatory response. In contrast, none of the Omicron-infected animals were found to harbor viral antigen in olfactory epithelium and brain (Fig. 6b). At 6 dpi, brain infection was confirmed by immunohistochemistry in five of the six Delta-infected mice, with generally widespread neuronal viral antigen expression (Fig. 6e); in two mice, it was accompanied by mild perivascular mononuclear infiltrates consistent with a non-suppurative encephalitis (39). In the nasal mucosa, in particular the olfactory epithelium, underlying nerve fibers were found to harbor viral antigen also at this stage. Again, the Omicron-infected mice did not show any viral antigen expression in nasal mucosa and brain (Fig. 6f).

After Ronapreve treatment, there was no evidence of viral antigen expression in the brain of any Delta-infected animal at 4 and 6 dpi (each *n* = 8; Fig. 6c and g); however, infected olfactory epithelial cells were detected at both time points, in four of the eight animals at 4 dpi, and in five of the eight animals at 6 dpi. Omicron-infected, Ronapreve-treated animals were negative for viral antigen in olfactory epithelium and brain at both time points (Fig. 6d and h), with the exception of the nasal mucosa of one mouse at 4 dpi. Detailed information on histological findings and viral antigen expression in the brains of individual animals is provided in Table S1.

### Ronapreve plasma concentrations

ELISAs were used to quantify casirivimab and imdevimab plasma concentrations on days 4 and 6 post-infection. The results outlined in Table 1 indicate comparable casirivimab and imdevimab plasma concentrations in both the Delta- and Omicron-inoculated mice at 4 and 6 dpi. Imdevimab plasma concentrations were higher in mice across both variants and time points investigated when compared to casirivimab. All samples from the vehicle control groups were shown to be below the limits of quantification.

**TABLE 1:** Mean Ronapreve (casirivimab and imdevimab) concentrations in mouse plasma at 4 and 6 dpi^*a*^

SARS-CoV-2 variant	Mean casirivimab plasma concentration ± SD (µg/mL)				Mean imdevimab plasma concentration ± SD (µg/mL)			
	4 dpi		6 dpi		4 dpi		6 dpi	
	Vehicle control	Ronapreve (400 µg)	Vehicle control	Ronapreve (400 µg)	Vehicle control	Ronapreve (400 µg)	Vehicle control	Ronapreve (400 µg)
Delta (B.1.617.2)	<LLQ	28.09 ± 0.38	<LLQ	28.74 ± 5.04	<LLQ	43.94 ± 3.12	<LLQ	41.80 ± 4.88
Omicron (B.1.1.529/BA.1)	<LLQ	27.76 ± 0.72	<LLQ	34.40 ± 4.29	<LLQ	46.90 ± 3.34	<LLQ	40.88 ± 0.93
Unpaired *t*-test	-	*P* = 0.315	-	*P* = 0.887	-	*P* = 0.127	-	*P* = 0.661

^*a*^ Mice were inoculated with 10^3^ PFU of SARS-CoV-2 Delta or Omicron and treated with a single intraperitoneal dose of the saline vehicle control or 400 µg Ronapreve in saline. Quantitative detection of each monoclonal antibody was completed separately using ELISA. <LLQ, below the lower limit of quantification.

## DISCUSSION

The current study made use of a reliable murine SARS-CoV-2 infection model to confirm and characterize the effect of Ronapreve on established infections with the Delta variant and confirm its ineffectiveness against BA.1 Omicron. Indeed, it provides increased certainty in the absence of effect against BA.1, complementing *in vitro* neutralization data for this variant (28, 41). However, it also confirms efficacy for the Delta variant and provides evidence that monoclonal antibodies might limit the virus spread into the brain when deployed against susceptible variants. In addition, our study indicates that the local application of virus binding monoclonal antibodies can induce inflammatory processes that might itself be of clinical relevance.

A very rapid comparable decline in body weight was observed in all groups of mice, at 2 dpi, different from previous studies that did not show consistent weight drop before 3 dpi (37–39). This early drop is likely a response to the invasiveness and additional handling associated with the intraperitoneal dosing. The weight gain toward day 3 moved body weights to levels observed in a previous study in which K18-hACE2 transgenic mice were infected with the same virus variants at the same dose but were not treated any further (35). By the end of the study, all but the saline control animals infected with the Delta variant had regained more weight. This observation is in agreement with the authors’ previous evaluation of the pathogenicity of these variants in K18 hACE2 transgenic mice (35, 38, 39). Mice infected with the Omicron variant generally carried less sub-genomic viral RNA than the Delta-variant-infected mice in both nasal turbinates and lungs at 4 and 6 dpi, which is consistent with previous reports (35). The histological and immunohistological results support this finding.

Consistent with the clinical evidence through body weight measurements, levels of sub-genomic RNA were reduced in both nasal turbinates and lung of mice infected with the Delta variant after Ronapreve treatment compared to controls, at both time points. This finding was complemented by the results of the histological and immunohistochemical examinations. Although the control group at 4 dpi in the majority exhibited widespread lung infection and associated alveolar damage with occasional vasculitis, the lungs of the Ronapreve-treated mice were either found unaltered and free of viral antigen, or exhibited a few small focal areas with alveolar damage and small patches of infected alveoli, but no evidence of vasculitis. This suggests that post-exposure Ronapreve treatment reduces pulmonary damage. Two days later, the difference between saline control and Ronapreve-treated mice was even greater. In the former, the lesions observed at 4 dpi were found to persist and the accompanying inflammatory response had intensified, resulting in larger consolidated areas and perivascular leukocyte infiltrates, with extensive multifocal viral antigen expression in large patches of alveoli. After Ronapreve treatment, a different inflammatory response and viral antigen expression pattern was observed. There were only a few very small patches with infected alveolar epithelial cells; however, viral antigen was also present in pneumocytes and macrophages within small, delineated focal macrophage dominated, i.e., granulomatous parenchymal infiltrates. Their proximity to and frequent continuity with identical focal infiltrates of vascular walls and the presence of viral antigen also within macrophages in these lesions indicate that they result from focal recruitment of macrophages into the parenchyma in response to virus. Ronapreve represents human antibodies that target the spike protein on the surface of SARS-CoV-2. After a single application, the antibodies will not have induced an immune response in the mice, instead they will likely have bound to the Fc receptors of the murine macrophages (42). Considering that the granulomatous reaction was not observed in the saline controls, it is likely that it represents the local response to antibody-opsonized virus that is phagocytosed by macrophages. A previous study that histologically examined the lungs of mice treated with a neutralizing antibody at 2 dpi as late as 21 days post-infection found unaltered lungs with only scarce lymphoid aggregates (33), indicating that these local processes can dissolve with time. Murine models are generally robust for identifying potential pathological effects of therapeutic interventions. The implications of these findings to clinical deployment of monoclonal antibodies are currently uncertain, but further robust assessment incorporating morphological measures in parallel to virological measures is warranted.

The immunohistochemical examination also revealed a further positive effect of the Ronapreve treatment. As expected (39), the Delta variant had already spread to the brain in some animals by day 4 and was found widespread in the brain at 6 dpi in mice that had received the saline control. At the later time point, it had induced a mild inflammatory response in some animals. After Ronapreve treatment, there was no evidence of viral antigen expression in the brain and no inflammatory change. Similarly, viral sgE RNA was below the level of detection. Because viral RNA detection was only undertaken in a small number of animals (*n* = 3), more extensive studies are warranted to further elucidate the effect of Ronapreve treatment on viral spread to the brain. These findings suggest that Ronapreve treatment post-exposure inhibits viral spread into the brain. Whether infection of the brain is completely blocked or only substantially reduced requires further investigations, particularly at the molecular level. In light of previous studies which showed that, in K18-hACE2 mice, the virus reaches the brain predominantly via the olfactory route (39, 40) and considering that Ronapreve treatment reduces viral loads in the nasal turbinates, it is probable that Ronapreve inhibits brain infection by reducing the risk of virus spread from the olfactory epithelium to the underlying nerves, then the olfactory bulb and into the brain.

Conversely, no significant impact of Ronapreve on sub-genomic RNA levels over 6 days was observed in mice infected with BA.1 Omicron, which is consistent with a lack of neutralization of this variant. The doses used in the current study were twofold higher than those for which virological efficacy was demonstrated in K18-hACE2 transgenic mice previously for other variants (27), which reinforces the conclusion that activity against BA.1 Omicron is ablated for Ronapreve. The immunohistochemical results further support the virological findings, as they indicate no or only mild reduction of viral antigen expression after Ronapreve treatment.

Curiously, the magnitude of the reduction in Delta sub-genomic RNA was lower in the present study than that reported for total RNA in a previous study, despite the higher dose (27). At the time of writing, the authors are unaware of other studies that have investigated the efficacy of Ronapreve for Delta in this model, but neutralization of Delta was not meaningfully compromised *in vitro* (14). Differences in the endpoint (sub-genomic versus total RNA measurements) make it difficult to draw firm conclusions from these observations but underscore the importance of *in vivo* evaluation of the efficacy of interventions against new and future variants.

Comparable casirivimab and imdevimab plasma concentrations were observed at 4 and 6 dpi in mice inoculated with either Delta or Omicron variants across the Ronapreve-administered groups. Ronapreve plasma concentrations in mice were consistent with those observed in a Phase 2 dose-ranging randomized controlled trial (RCT), which demonstrated virological efficacy in treatment following a single 300 or 600 mg intravenous dose (43). This study utilized intraperitoneal administration of Ronapreve in mice. However, comparable virological efficacy was observed in the RCT across all Ronapreve doses (300–2,400 mg intravenous) and routes of administration (600–1,200 mg subcutaneous) investigated. It was noted that the trial was conducted before the emergence of the Omicron variant and demonstrates dose-ranging virological efficacy in known susceptible variants (43). Further preclinical evaluation of monoclonal antibody penetration into target tissues like the lung would improve the characterization of compartments associated with SARS-CoV-2 replication.

The experimental design employed here reflects treatment whereby the intervention was applied subsequent to the inoculation of the animals with virus. Several other studies that have sought to assess continued efficacy of monoclonal antibodies against later Omicron sub-lineages have utilized prophylactic designs where the antibody is administered prior to inoculation of the animals with virus (44–46). Because of the differences in viral load when the intervention is introduced, it is well established for antiviral interventions that the bar is much higher to achieve efficacy in treatment than it is for prophylaxis. The data presented here clearly demonstrate that *in vivo* designs reflecting the intended treatment use case are achievable and demonstrate efficacy for monoclonal antibodies against susceptible variants. Extreme caution should be taken when interpreting *in vivo* data from prophylactic designs while making an assessment of the likely continued efficacy in treatment. Where animal data are used to support candidacy of interventions, *in vivo* studies should be designed to be reflective of the intended use case in humans.

## MATERIALS AND METHODS

### Materials

Materials were purchased and used as received without further purification: chloroform, isopropanol, ethanol, phosphate-buffered saline (PBS), and nuclease-free water were purchased from Fisher Scientific (UK). Male K18-hACE2 mice were purchased from Charles River (France). Ronapreve (casirivimab and imdevimab) was kindly provided by Roche (Switzerland). TRIzol, GlycoBlue, Phasemaker tubes, and TURBO DNA-free kit were purchased from Fisher Scientific (UK). GoTaq Probe 1-Step RT-qPCR System was purchased from Promega (USA). SARS-CoV-2 (2019nCoV) CDC qPCR Probe Assay was purchased from IDT (USA). Precellys CKmix lysing tubes were purchased from Bertin Instruments (France). For immunohistology, a rabbit anti-SARS-CoV nucleoprotein antibody was purchased from Rocklands; the peroxidase blocking buffer, the Envision+System HRP Rabbit, and the diaminobenzidine were from Agilent/Dako. All other chemicals and reagents were purchased from Merck (UK) and were used as received, unless stated otherwise.

### Virus isolates

The Delta variant (B.1.617.2) hCoV-19/England/SHEF-10E8F3B/2021 (GISAID accession number EPI_ISL_1731019) was kindly provided by Prof. Wendy Barclay, Imperial College London, London, UK, through the Genotype-to-Phenotype National Virology Consortium (G2P-UK). Sequencing confirmed it contained the spike protein mutations T19R, K77R, G142D, Δ156-157/R158G, A222V, L452R, T478K, D614G, P681R, D950N. The Omicron variant (B.1.1.529/BA.1) isolate M21021166 was originally isolated by Prof. Gavin Screaton, University of Oxford (29), UK, and then obtained from Prof. Wendy Barclay, Imperial College London, London, UK, through G2P-UK. Sequencing confirmed it contained the spike protein mutations A67V, Δ69-70, T95I, G142D/Δ143-145, Δ211/L212I, ins214EPE, G339D, S371L, S373P, S375F, K417N, N440K, G446S, S477N, T478K, E484A, Q493R, G496S, Q498R, N501Y, Y505H, T547K, D614G, H655Y, N679K, P681H, N764K, A701V, D796Y, N856K, Q954H, N969K, L981F. The titers of all isolates were confirmed on Vero E6 cells, and the sequences of all stocks were confirmed.

### Animal studies

All work involving SARS-CoV-2 was performed at containment level 3 by staff equipped with respirator airstream units with filtered air supply. Prior to the start of the study, all risk assessments and standard operating procedures were approved by the University of Liverpool Biohazards Sub-Committee and the UK Health and Safety Executive.

All animal studies were conducted in accordance with the UK Home Office Animals Scientific Procedures Act (47). Additionally, all studies were approved by the local University of Liverpool Animal Welfare and Ethical Review Body and were performed under the UK Home Office Project License PP4715265. Animal studies complied with the ARRIVE guidelines. Male mice (20–30 g) carrying the human ACE2 gene under the control of the keratin 18 promoter [K18-hACE2; formally B6.Cg-Tg(K18-ACE2)2Prlmn/J] were housed in individually ventilated cages with environmental enrichment under SPF barrier conditions and a 12-hour light/dark cycle at 21°C ± 2°C. Free access to food and water was provided at all times.

Mice were randomly assigned into groups and acclimatized for 7 days. Mice in each group were anesthetized under 3% isoflurane and inoculated intranasally with 100 µL of either 10^3^ PFU of SARS-CoV-2 Delta variant (B.1.617.2) or Omicron variant (B.1.1.529) in PBS. After 24 hours, mice from each group were treated with a single dose (100 µL) of either the saline control or 400 µg Ronapreve, diluted in saline, via intraperitoneal (IP) injection. All animals were weighed and monitored daily throughout the experiment. At 4 and 6 days following infection, groups of mice were sacrificed via a lethal IP injection of pentobarbitone, followed by cardiac puncture and immediate exsanguination from the heart. Animals were immediately dissected, and the right lung as well as fragments from the nasal turbinates was collected and frozen at −80°C for RNA extraction. The left lung lobe and the head were fixed in 10% buffered formalin for 48 hours and then stored in 70% ethanol until processing for histological and immunohistological examination.

In a separate study, utilizing a comparable experimental design for infection with the SARS-CoV-2 Delta variant and the Ronapreve treatment approach, viral replication in mouse lung and brain was quantified using qRT-PCR. At 9 dpi, mice were sacrificed via a lethal IP injection of pentobarbitone, followed by cardiac puncture and immediate exsanguination from the heart. Animals were immediately dissected, the lung and brain were collected and stored at −80°C prior to homogenization for RNA extraction.

### Quantification of viral RNA

RNA isolation from lung, nasal turbinate, and brain samples, RNA quantification, and DNAse treatment have been detailed previously (30).

The viral RNA derived from the lung, nasal turbinate, and brain samples was quantified using a protocol for quantifying the SARS-CoV-2 sub-genomic E gene RNA (sgE) (48) using the GoTaq Probe 1-Step RT-qPCR System (Promega).

Quantification of SARS-CoV-2 sgE RNA was completed utilizing primers and probes previously described elsewhere (48), which were used at 400 and 200 nM, respectively (IDT), using the GoTaq Probe 1-Step RT-qPCR System (Promega). Quantification of 18S RNA utilized previously described primers and probe sequences (49), which were used at 300 and 200 nM, respectively (IDT), using the GoTaq Probe 1-Step RT-qPCR System (Promega). Methods for the generation of the 18S and sgE RNA standards have been outlined previously (50). Both PCR products were serially diluted to produce standard curves in the range of 5 × 10^8^ – 5 copies/reaction via a 10-fold serial dilution. DNAse-treated RNA at 20,000 ng/mL or dH_2_O was added to appropriate wells producing final reaction volumes of 20 µL. The prepared plates were run using a qTOWER³ Real-Time PCR Detector (Analytik Jena). Thermal cycling conditions have been detailed previously (30). The sgE data were normalized to 18S data for subsequent quantitation.

### Statistical analysis

An unpaired, two-tailed, *t*-test was used to compare the differences in lung, nasal turbinate, and brain viral RNA between the control (saline) and Ronapreve treatment groups. A *P* value of ≤0.05 was considered statistically significant. All statistical analyses were completed using Prism v.10 (GraphPad, USA).

### Histological and immunohistochemical analyses

The fixed left lung was routinely embedded in paraffin wax. Heads were sawn longitudinally in the midline using a diamond saw (Exakt 300; Exakt), and the brain was left in the skull. Heads were gently decalcified in RDF (Biosystems) twice for 5 days, at room temperature (RT) and on a shaker, then both halves were embedded in paraffin wax. Consecutive sections (3–5 µm) were prepared and stained with hematoxylin eosin (HE) for histological examination or subjected to immunohistochemical staining to detect SARS-CoV-2 antigen (performed in an autostainer; Agilent), using the horseradish peroxidase (HRP) method and rabbit anti-SARS-CoV nucleocapsid protein as previously described (39). Briefly, sections were deparaffinized and rehydrated through graded alcohol. Antigen retrieval was achieved by a 20-minute incubation in citrate buffer (pH 6.0) at 98°C in a pressure cooker. This was followed by incubation with the primary antibody (diluted 1:3,000 in dilution buffer; Dako) overnight at 4°C, a 10-minute incubation at RT with peroxidase blocking buffer (Agilent), and a 30-minute incubation at RT with Envision+System HRP Rabbit (Agilent). The reaction was visualized with diaminobenzidin (DAB; Dako) for 10 minutes at RT. After counterstaining with hematoxylin for 2 seconds, sections were dehydrated and covered with glass coverslips. In selected animals (see Table S1), lungs were also stained for CD3 (T cell marker), CD45R/B220 (B cell marker), and Iba1 (macrophage marker), as previously described (39).

### Quantification of Ronapreve in mouse plasma

ELISA was used for the quantitative detection of casirivimab and imdevimab in mouse plasma. Briefly, the terminal cardiac bleed from each animal was collected in lithium heparin tubes (Sarstedt, Germany) and was centrifuged for 15 minutes at 1,000 × *g*. The isolated plasma was inactivated with 0.5% vol/vol Triton X-100 in dH_2_O and was stored at −80°C prior to analysis.

Subsequently, the plasma samples were diluted 1:100 with dH_2_O, and each target was quantified separately using casirivimab and imdevimab ELISA kits (abx395207 and abx395208, Abbexa, UK) according to the manufacturer’s instructions. Briefly, a standard curve was prepared ranging from 2,000 to 15.6 ng/mL by serial dilution. Each standard, blank (standard diluent), and sample was prepared in duplicate, added to the 96-well ELISA microplates, and processed according to the manufacturer’s instructions. Following incubation, the optical density (OD) (450 nm) of each well was measured using a BioTek Synergy microplate reader (Agilent, USA). An inverse correlation is observed between each monoclonal antibody concentration and the measured OD. Average sample concentrations were calculated based on their OD values using the standard curves. Sample concentrations were determined using Prism v.10 (GraphPad, USA).

## Data Availability

The authors report that all data supporting the findings of this study are presented within the paper and supplementary material.

## SUPPLEMENTAL MATERIAL

Table S1 spectrum.03916-23-s0001.docx Relevant histological changes and SARS-CoV-2 nucleoprotein expression in K18-hACE2 mice.

ASM does not own the copyrights to Supplemental Material that may be linked to, or accessed through, an article. The authors have granted ASM a non-exclusive, world-wide license to publish the Supplemental Material files. Please contact the corresponding author directly for reuse.
